# C3G knock-down enhances migration and invasion by increasing Rap1-mediated p38α activation, while it impairs tumor growth through p38α-independent mechanisms

**DOI:** 10.18632/oncotarget.9911

**Published:** 2016-06-07

**Authors:** Neibla Priego, María Arechederra, Celia Sequera, Paloma Bragado, Ana Vázquez-Carballo, Álvaro Gutiérrez-Uzquiza, Víctor Martín-Granado, Juan José Ventura, Marcelo G. Kazanietz, Carmen Guerrero, Almudena Porras

**Affiliations:** ^1^ Departamento de Bioquímica y Biología Molecular II, Facultad de Farmacia, Universidad Complutense de Madrid, Madrid, Spain; ^2^ Instituto de Investigación Sanitaria del Hospital Clínico San Carlos (IdISSC), Madrid, Spain; ^3^ Institut d'Investigacions Biomediques August Pi i Sunyer (IDIBAPS), Barcelona, Spain; ^4^ Centro de Investigación del Cáncer, IBMCC, Departamento de Medicina, Facultad de Medicina, Universidad de Salamanca, Instituto de Investigaciones Biomédicas de Salamanca (IBSAL), Salamanca, Spain; ^5^ Translational Cell and Tissue Research, Department of Imaging and Pathology, Leuven University, Leuven, Belgium; ^6^ Department of Systems Pharmacology and Translational Therapeutics, Perelman School of Medicine, University of Pennsylvania, Philadelphia, PA, USA; ^7^ Present address: Department of Cancer Biology, Biomedical Research Building II/III, School of Medicine, University of Pennsylvania, Philadelphia, PA, USA

**Keywords:** C3G, p38 MAPK, Rap1, migration, tumorigenesis

## Abstract

C3G, a Guanine nucleotide Exchange Factor (GEF) for Rap1 and R-Ras, has been shown to play important roles in development and cancer. Previous studies determined that C3G regulates cell death through down-regulation of p38α MAPK activity. Here, we found that C3G knock-down in MEFs and HCT116 cells promotes migration and invasion through Rap1-mediated p38α hyper-activation. These effects of C3G were inhibited by Rap1 knock-down or inactivation. The enhanced migration observed in C3G depleted HCT116 cells was associated with reduction in E-cadherin expression, internalization of ZO-1, actin cytoskeleton reorganization and decreased adhesion. We also found that matrix metalloproteases MMP2 and MMP9 are involved in the pro-invasive effect of C3G down-regulation. Additionally, our studies revealed that both C3G and p38α collaborate to promote growth of HCT116 cells *in vitro* and *in vivo*, possibly by enhancing cell survival. In fact, knocking-down C3G or p38α individually or together promoted cell death *in vitro*, although only the double C3G-p38α silencing was able to increase cell death within tumors. Notably, we found that the pro-tumorigenic function of C3G does not depend on p38α or Rap1 activation. Altogether, our studies uncover novel mechanisms by which C3G controls key aspects of tumorigenesis.

## INTRODUCTION

C3G (Crk SH3-domain-binding guanine-nucleotide-releasing factor) is a Guanine nucleotide Exchange Factor (GEF) for Rap1 and R-Ras proteins [1–3] that is essential for embryonic development due to its function in integrin-mediated cellular adhesion and migration [3].

C3G has been shown to regulate cell migration in different ways depending on the context. For example, C3G deficiency enhances migration in mouse embryonic fibroblasts (MEFs), while it impairs cell adhesion and delays cell spreading [3–4]. In contrast, the absence of C3G or a hypermorphic C3G mutation leads to impaired cortical [5] and sympathetic preganglionic neurons migration [6], respectively. On the other hand, C3G overexpression leads to opposite outcomes in different cell types, as it increases migration of glomerulal epithelia cells in glomerulonephritis [7], while decreasing migration in highly invasive breast carcinoma cells [8]. Several lines of evidence suggest that C3G can exert effects through mechanisms that are independent of its GEF activity [9–11].

The function of C3G in cell migration has been described to be mediated, at least in part, by its main target, Rap1 [3, 12–13]. C3G overexpression can also promote c-Abl-induced filopodia formation through mechanisms independent of its catalytic activity [14]. C3G is known to regulate cell-cell interactions [13, 15], where E-cadherin plays a key role [13]. Mechanistically, it has been found that C3G binds intracellular E-cadherin, and this leads to activation of Rap-1 and E-cadherin translocation [13].

The function of C3G in human cancer has been a subject of controversy for many years. In mouse fibroblasts, C3G can act as a tumor suppressor gene, as it prevents malignant transformation induced by several oncogenes [9–10,16]. Accordingly, C3G expression is reduced in cervical squamous cell carcinoma [17]. In contrast, elevations in C3G expression have been found in human non-small-cell lung cancer [18]. The expression of the p87C3G isoform in chronic myeloid leukemia (CML) cells has been causally associated with disease development [19]. Recent data also suggested that C3G, acting through Rap1, induces invasion of epithelial ovarian cancer cells and promotes the secretion of matrix metalloproteases MMP2 and MMP9 [20]. In colon carcinoma, the C3G gene is frequently demethylated [21], but it remains to be determined whether this epigenetic modification is associated with changes in C3G expression or if it plays any relevant role in the progression of this cancer.

There is extensive evidence for the involvement of p38α MAPK in cancer. Like C3G, p38α has been shown to act also both as a tumor suppressor or tumor promoter depending on the type of cancer and tumor stage [22]. p38α was found to inhibit tumor initiation by promoting cell cycle arrest and/or inducing apoptosis [22–24]. In contrast, increased levels of phosphorylated (active) p38α have been correlated with malignancy in various cancer types [22], such as head and neck carcinoma [25]. It has been also reported that at late stages of tumor development, p38α can promote cell survival [22, 26], migration and invasion, thereby contributing to the metastatic dissemination of cancer cells [22]. Accordingly, p38α MAPK is required for cell migration in MEFs [27], and several cancer cell lines [22, 28]. Moreover, p38α negatively regulates cell adhesion in embryonic stem cells [29] and cardiomyocyte-derived cell lines [30], which would result in an enhanced migratory phenotype. We have recently reported that p38α promotes growth of HCT116 colon cancer cells *in vitro* and in nude mice, as well as positively regulates migration and invasion [27]. In agreement with these findings, recent studies highlighted a fundamental role for p38α in promoting cell proliferation and survival in a mouse model of colitis-associated tumor induction [31].

Using the CML cell line K562 and MEFs deficient in p38α and/or C3G, we have previously reported that C3G, through down-regulation of p38α activity, positively or negatively regulates apoptosis, depending on the stimulus [32–33]. C3G and p38α also display antagonistic roles in the regulation of focal adhesion (FA) complex formation in K562 cells [34]. Based on these previous findings, in the present study we wished to determine if p38α could also mediate the effect of C3G on cell migration and invasion. In addition, we investigated if the C3G/p38α pathway could be potentially involved in tumor growth. Our results revealed that C3G inhibits cell migration and invasion by interfering with Rap1-mediated p38α activation. On the other hand, both C3G and p38α are capable of promoting colon carcinoma tumor growth mainly through different mechanisms.

## RESULTS

### C3G silencing increases migration and invasion of MEFs through a mechanism dependent on p38α MAPK

**Figure 1:**
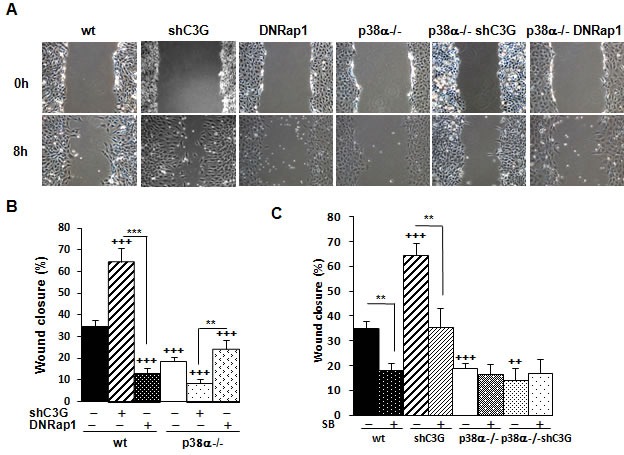
C3G knock-down enhances migration of MEFs through a mechanism dependent on p38α Wound healing assay. MEFs (wt and p38α−/−, with (shC3G) or without C3G knock-down or expression of DNRap1 (DNRap1)) were maintained in the absence of serum and allowed to migrate. **A.** Representative images from phase contrast microscope after 0 and 8h of migration. **B.** and **C.** Histograms show the mean ± S.E.M. of the percentage of wound closure (*n* = 4). ^++^*p* < 0.01 and ^+++^*p* < 0.001, *versus* wt; **p< 0.01; ***p < 0.001, compared as indicated. **C.** Effect of p38α/β inhibition with the chemical inhibitor, SB203580 (10μM), on cell migration.

In the first set of experiments, we took advantage of loss-of-function approaches to establish the involvement of C3G and p38α in MEF cell motility. As shown in Figure 1A and 1B, wound healing assays revealed that C3G knock-down enhanced cell migration in wt MEFs, but not in p38α−/− cells. Moreover, time-lapse microscopy analysis showed that C3G knock-down MEFs expressing p38α lost cell-cell interactions, escaped from the wound border, and moved away (Suppl. Videos). In contrast, MEFs lacking p38α moved slowly and collectively, maintaining cell-cell interactions, and in these cells, C3G knock-down has not a major effect.

To determine if C3G was acting through its main target, Rap1, we evaluated the effect of a dominant negative Rap1 (DNRap1) construct using a MEFs cell line previously established, where Rap1-GTP levels are very low [33]. Figure 1A and 1B show a reduction in migration in wt cells expressing DNRap1, which correlates with the reduction in phospho-p38α levels (Supplementary Figure 1). In p38α−/− MEFs, no significant effect was observed. To further demonstrate the relevance of p38α, the effect of the selective p38α/β inhibitor SB203580 was examined. Treatment with this p38 inhibitor prevented the enhancing effect of C3G knock-down on migration in wt MEFs and decreased the migratory ability of non-silenced cells (Figure 1C and Supplementary Figure 2). These results strongly indicate that p38α mediates the pro-migratory effect caused by C3G silencing.

**Figure 2:**
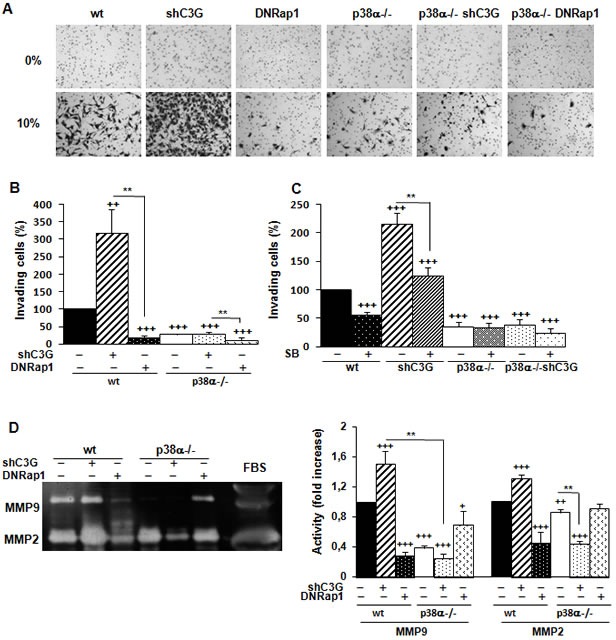
C3G silencing increases the invasive capacity of MEFs by a mechanism mediated by p38α and dominant negative Rap1 impairs invasion MEFs (wt and p38α−/−, with (shC3G) or without C3G knock-down or expression of DNRap1 (DNRap1)) were maintained in the absence of serum for the last 24h. **A.**, **B.** and **C.** Invasion through Matrigel using FBS (10%) as chemoattractant. **A.** Representative images of invading cells after staining with crystal violet (phase contrast microscope). **B.** and **C.** Histograms show the mean value ± S.E.M. of the percentage of invading cells (*n* = 4). ^++^*p* < 0.01 and ^+++^*p*< 0.001, *versus* wt; **p < 0.01, compared as indicated. **C.** Effect of p38α/β inhibition with SB203580 (10μM) on cell invasion. **D.** Zymographic analysis of MMP2 and MMP9 activities using gelatin as the substrate and FBS as a control. Representative zymogram (left panel). Histogram (right panel) showing the mean ± S.E.M. of the densitometric analysis of gelatinase areas expressed as fold increase of the control value (*n* = 6). ^+^*p* < 0.05,^++^*p* < 0.01 and ^+++^*p* < 0.001, *versus* wt; **p < 0.01, compared as indicated.

Next, we evaluated the effect of C3G on invasion. C3G knock-down markedly enhanced invasion of wt MEFs through Matrigel, but not that of p38α−/− cells (Figure 2A and 2B). Cells lacking p38α had a very low invasive capacity. Moreover, the expression of the DNRap1 impaired invasion of wt MEFs. These results indicate that the increased invasion induced by C3G depletion requires p38α activation. This was further supported by the inhibitory effect of SB203580 on the invasive effect on Matrigel (Figure 2C) and collagen (data not shown) caused by C3G knock-down.

MMPs are relevant for extracellular matrix degradation during migration and invasion [35–36], and some of them are regulated by p38α, such as MMP2 and MMP9 [27, 37]. As shown in Figure 2D, MMP2 and MMP9 activities were higher in wt than in p38α−/− MEFs, and they were further increased upon C3G knock-down in wt MEFs. In contrast, activities of these MMPs decreased in wt cells expressing DNRap1. In addition, treatment with SB203580 markedly reduced MMP9 activity in wt cells, with or without C3G silencing, and slightly inhibited MMP2 activity (Supplementary Figure 3). These data suggest the involvement of MMP2 and MMP9 in the pro-invasive effect caused by C3G knock-down as well as in the inhibitory effect of DNRap1.

### C3G knock-down enhances migration and invasion of HCT116 cells through a mechanism dependent on p38α

**Figure 3:**
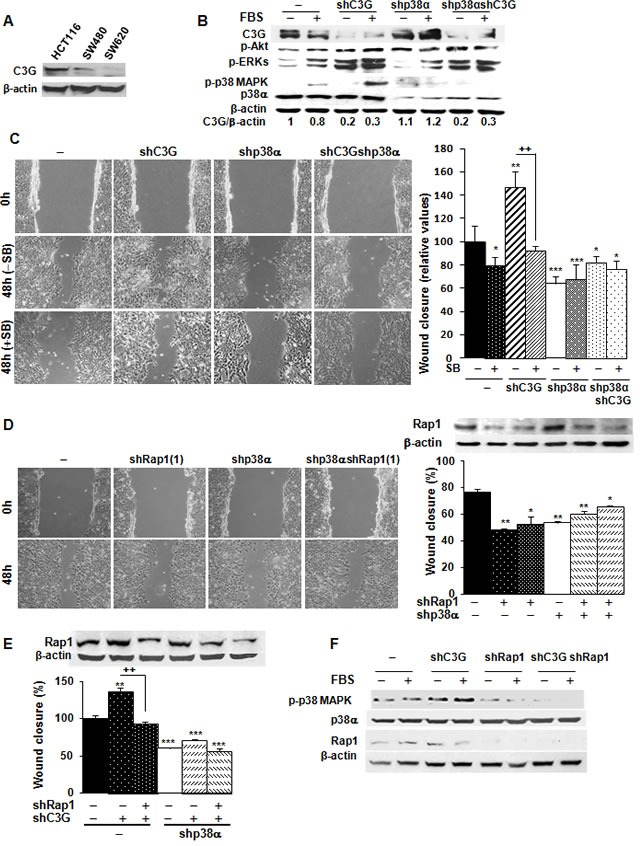
C3G knock-down enhances migration of HCT116 cells by increasing Rap1-mediated p38α activation HCT116 (non-silenced (−) and p38α knock-down (shp38α); with (shC3G) or without C3G knock-down; with (shRap1(1) and shRap1(2)) or without Rap1 knock-down), SW480 and SW620 cells were used. **A.** C3G protein expression in HCT116, SW480 and SW620 cells analyzed by Western-blot and normalized with β-actin. **B.** Western-blot analysis of P-Akt, P-ERKs and P-p38 MAPK levels normalized with β-actin. p38α and C3G were measured as a control of their expression. **C.** Wound healing assays. Left panel, representative images from phase contrast microscope after 0 and 48h of migration, in the absence or presence of p38α/β inhibitor, SB203580 (10μM). Right panel, the histogram shows the mean ± S.E.M. of the percentage of wound closure (*n* = 4). *p < 0.05,**p < 0.01; ***p < 0.001, *versus* non-silenced cells; ^++^*p* < 0.01, compared as indicated. **D.** Effect of Rap1 knock-down using two different shRNAs. Left panel, representative images from phase contrast microscope after 0 and 48h of migration upon transient Rap1 silencing using one of the shRNAs against Rap1. Right panel, Rap1 protein expression analyzed by Western-blot and normalized with β-actin (upper site) and histogram (lower site) showing the mean ± S.E.M. of the percentage of wound closure using the two different shRNAs against Rap1 (*n* = 4). *p < 0.05,**p < 0.01 *versus* non-silenced cells. **E.** Effect of C3G and Rap1 double knock-down on wound healing closure. Rap1 protein expression analyzed by Western-blot and normalized with β-actin (upper panel) and histogram (lower panel) showing the mean ± S.E.M. of the percentage of wound closure (*n* = 3). **p < 0.01 and ***p < 0.001, *versus* non-silenced cells; ^++^*p* < 0.01, compared as indicated. **F.** Analysis of p38α activation. Western-blot analysis of P-p38 MAPK, p38α and Rap1 levels normalized with β-actin in cells expressing p38α.

C3G is known to regulate migration, invasion, as well as the tumorigenic activity of various cancer cell types [8, 15, 17–20]. However, the functional relevance of C3G in colon carcinoma has not been characterized. First, we examined C3G protein levels in human colon carcinoma cell lines with different invasive capacities: HCT116 cells (low invasive ability), SW480 and SW620 cells (high invasive ability). As shown in Figure 3A, the highest C3G expression levels were found in the least invasive cell line, HCT116 cells, thus suggesting an inverse correlation between C3G protein levels and colon carcinoma cells invasive capacity. Next, we determined the effect of C3G down-regulation on the migratory and invasive capacities of HCT116 cells. Notably, knocking-down C3G in parental cells led to a significant up-regulation of the levels of phospho-p38α, phospho-Akt and phospho-ERKs in response to serum (Figure 3B), as previously described in MEFs [33]. Hence, we next determined the effect of C3G knock-down on migration and whether this effect was dependent on p38α. Figure 3C shows that C3G silencing enhanced migration in HCT116 cells expressing p38α, but not in those subjected to p38α depletion. Moreover, inhibition of p38α/β with SB203580 prevented the enhancement of migration induced by C3G knock-down in cells expressing p38α (Figure 3C) and reduced the migratory capacity of non-silenced cells. These results suggest that C3G inhibits migration through p38α inhibition.

To assess if the effects of C3G were mediated by Rap1, Rap1 was knocked-down in parental HCT116 cells using two different shRNAs. Figure 3D shows that Rap1 levels were significantly reduced by both shRNAs, which reduced migration. This suggests that Rap1 does not mediate C3G effects on migration. To confirm this, Rap1 was transiently knocked-down in C3G silenced HCT116 cells. Surprisingly, Rap1 knock-down prevented the increase in migration induced by C3G knock-down in cells expressing p38α (Figure 3E), in a similar way that p38α/β inhibition did. Accordingly, phospho-p38 MAPK levels markedly decreased upon Rap1 knock-down in cells expressing p38α, either with or without C3G silencing (Figure 3F). These results indicate that in the absence of C3G, compensatory mechanisms are activated and other Rap1 GEFs could possibly activate Rap1. This would lead to increase p38α activation and consequently, enhance migration.

**Figure 4:**
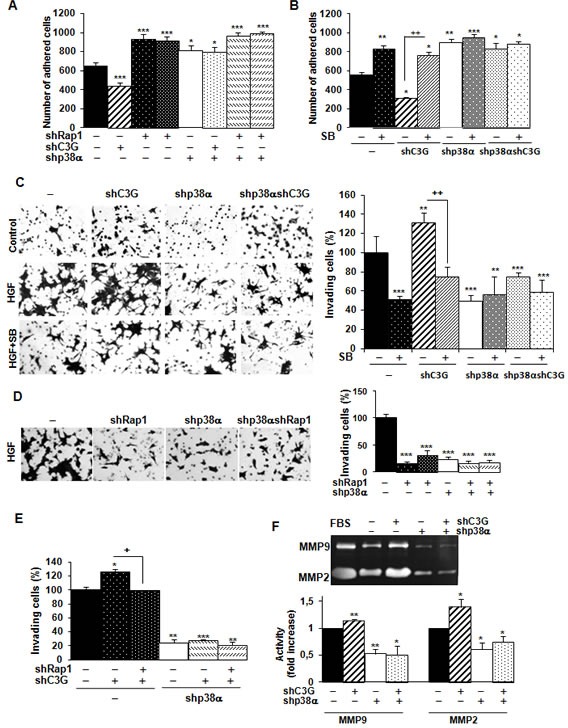
C3G silencing promotes invasion of HCT116 cells by a mechanism mediated by p38α, but not by Rap1 Effect of C3G knock-down on adhesion. HCT116 cells (non-silenced (−) and p38α knock-down (shp38α); with (shC3G) or without C3G knock-down; with (shRap1) or without Rap1 knock-down) were maintained in the presence (adhesion assays) or absence (invasion assays) of serum and in the presence or absence of the p38α/β inhibitor, SB203580 (5μM). **A.** and **B.** Adhesion assays. Histograms show the mean ± S.E.M. of the number of adhered cells 1h after platting. **C.** and **D.** Invasion through Matrigel using HGF as chemoattractant. Left panels, representative images of invading cells after staining with crystal violet (phase contrast microscope). Right panels, histograms showing the mean value ± S.E.M. of the percentage of invading cells referred to non-silenced (100%) (*n* = 4). **p < 0.01, ***p < 0.001, *versus* non-silenced cells; ^++^*p* < 0.01, compared as indicated. **E.** Effect of C3G and Rap1 double knock-down on invasion through Matrigel. The histogram shows the mean value ± S.E.M. of the percentage of invading cells referred to non-silenced (100%).*n* = 3). *p < 0.05, **p < 0.01, ***p < 0.001, *versus* non-silenced cells; ^+^*p* < 0.05, compared as indicated. **F.** Zymographic analysis of MMP2 and MMP9 activities using gelatin as the substrate and FBS as a control. Representative zymogram (upper panel). Histogram (lower panel) showing the mean ± S.E.M. of the densitometric analysis of gelatinase areas expressed as fold increase of the control value (*n* = 4). *p < 0.05, **p < 0.01, *versus* non-silenced cells.

We also found that C3G knock-down reduced adhesion of HCT116 cells expressing p38α (Figure 4A), which was prevented by Rap1 knock-down (Supplementary Figure 4). Both single p38α and Rap1 depletion increased adhesion, as also observed upon inhibition of p38α/β with SB203580 (Figure 4B). Indeed, C3G knock-down cells expressing p38α showed the lowest adhesion, which might favor migration. Accordingly, invasion of these cells through Matrigel using HGF as chemoattractant was increased (Figure 4C), and this effect was dependent on p38α activation, as p38α knock-down or SB203580 impaired the invasive response (Figure 4C). Similarly, Rap1 knock-down abrogated invasion of parental cells (Figure 4D) and impaired the pro-invasive effect caused by C3G silencing (Figure 4E).

MMP2 and MMP9 activities were higher in HCT116 cells expressing p38α relative to p38α knock-down cells (Figure 4F). These differences were further increased upon C3G silencing (Figure 4F). In contrast, Rap1 knock-down decreased MMP2 and MMP9 activities in cells expressing p38α (Supplementary Figure 5). Other MMPs reported to be important for invasion of colon carcinoma cells (MMP7, MMP10 and MMP13) did not show any significant change in expression as a consequence of C3G and/or p38α knock-down, as determined by RT-qPCR (data not shown).

### Mechanisms involved in the regulation of migration by C3G

Cell migration involves the re-organization of F-actin cytoskeleton and the generation of structures such as ruffles, filopodia and lamellipodia [38]. These effects can be regulated by p38α [28]. Changes in the expression and/or subcellular localization of cell-cell contact proteins also occur during migration [38–40]. To gain further insights into the mechanisms involved in the regulation of migration and invasion by C3G, we analyzed F-actin cytoskeleton organization, as well as the levels and subcellular localization of relevant cell junction proteins, namely E-cadherin and ZO-1.

**Figure 5:**
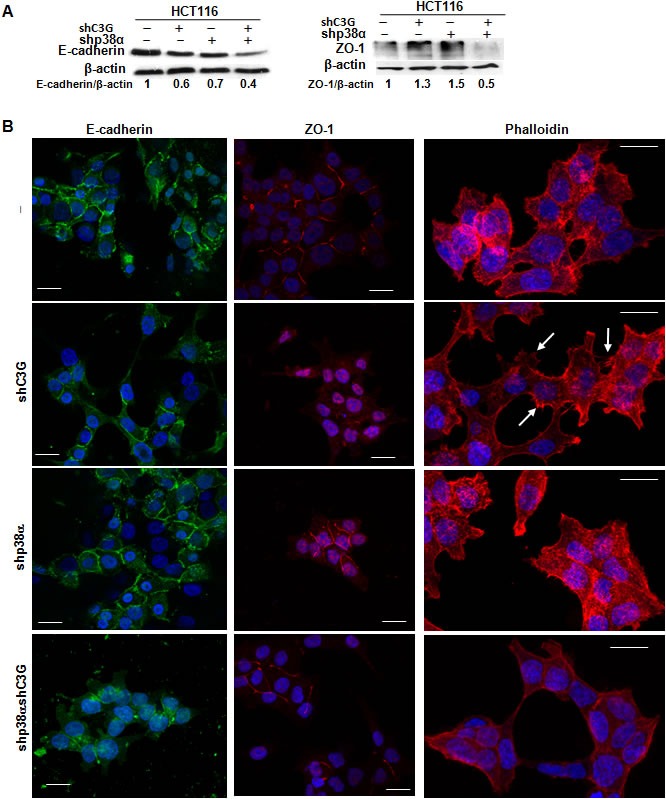
Effect of C3G knock-down on actin-organization, E-cadherin and ZO-1 expression and their subcellular localization in HCT116 cells. Function of p38α MAPK HCT116 cells (non-silenced (−) and p38α knock-down (shp38α); with (shC3G) or without C3G knock-down) were used. **A.** Representative western-blot analysis of E-cadherin and ZO-1 levels normalized with β-actin. The ratios E-cadherin/β-actin and ZO-1/β-actin of the densitometric analyses are shown. **B.** Representative confocal microscopy images of E-cadherin, ZO-1 and actin staining, using specific antibodies or phalloidin, respectively. Scale bar = 20μm. Arrows indicates the presence of filopodia.

E-cadherin is known to interact with C3G, which participates in the maturation of E-cadherin-based cell-cell contacts [41–42]. E-cadherin levels were substantially reduced in HCT116 cells subjected to C3G knock-down (Figure 5A), with or without p38α depletion. Silencing p38α also led to E-cadherin down-regulation, and indeed, the largest reduction in E-cadherin levels was observed in cells with double C3G-p38α knock-down. As this reduction in E-cadherin did not correlate with the migratory and invasive capacity of the cells (see Figure 3C and 4C), we analyzed changes in E-cadherin subcellular localization by confocal microscopy. In all cases, E-cadherin was primarily located in the plasma membrane (Figure 5B), although it was partially internalized in cells subject to dual knock-down.

Although no significant changes in ZO-1 total protein levels could be observed upon C3G knock-down in cells expressing p38α (Figure 5A), this protein was found to be internalized (Figure 5B). In contrast, in p38α silenced HCT116 cells, ZO-1 was mainly present in the plasma membrane, as also observed in cells subjected to double C3G/p38α knock-down, where ZO-1 total levels were reduced (Figure 5A and 5B). These data suggest that cell-cell contacts, particularly tight junctions, are partially disrupted in C3G knock-down HCT116 cells expressing p38α, which would result in enhanced migration.

We also examined F-actin cytoskeleton organization by confocal microscopy. As shown in Figure 5B, the presence of filopodia and lamellipodia was highly noticeable in C3G knock-down HCT116 cells expressing p38α (see arrows), an effect that correlates with the enhanced migratory capacity of these cells.

### C3G and p38α MAPK promote HCT116 cells foci formation and *in vivo* tumor growth through independent mechanisms

**Figure 6:**
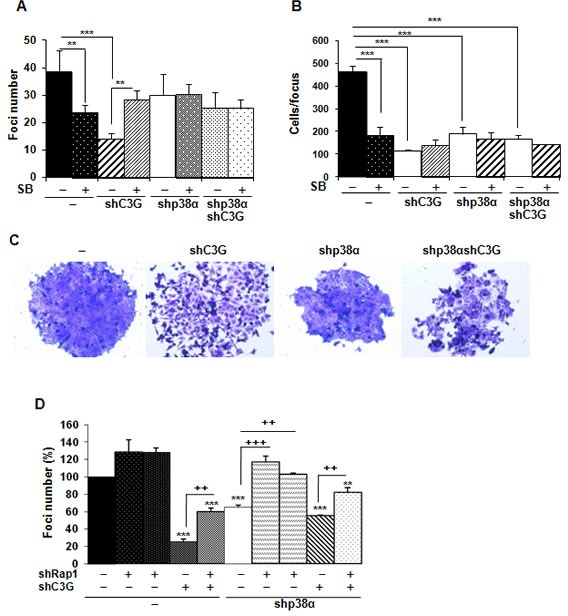
C3G and p38α MAPK, but not Rap1, promote anchorage dependent growth of HCT116 cells HCT116 cells (non-silenced (−) and p38α knock-down (shp38α); with (shC3G) or without C3G knock-down; with (shRap1(1) and shRap1(2)) or without Rap1 knock-down) were used. The effect of p38α/β inhibition by SB203580 (10μM) was also assessed. Anchorage dependent growth assays. Histograms show the mean ± S.E.M. of foci number **A.** and **D.** or the number of cells per focus (*n* = 4) **B.**. **A.** and **B.** *p < 0.05,**p < 0.01,***p < 0.001, compared as indicated. **C.** Representative images of individual foci. **D.** Effect of C3G, Rap1 and C3G-Rap1 double knock-down on foci formation. **p < 0.01, ***p < 0.001, *versus* non-silenced cells; ^++^*p* < 0.01 and ^+++^*p* < 0.001, compared as indicated (*n* = 3).

Anchorage-dependent growth assays revealed a reduction in the number of foci in C3G knock-down cells, which was more prominent in cells expressing p38α (Figure 6A). The number of foci was also reduced as a consequence of p38α silencing. These results suggest that both C3G and p38α promote foci formation probably through distinctive pathways. Moreover, inhibition of p38α with SB203580 reduced the number of foci in non-silenced cells, thus recapitulating the effect of p38α knock-down (Figure 6A). Surprisingly, in C3G depleted cells, the foci number was increased in response to SB203580 treatment, while having no effect on double C3G-p38α knock-down cells. To gain further insights into the function of C3G and p38α in foci formation, the number of cells per focus and its morphology was assessed. As shown in Figure 6B, both C3G and p38α knock-down decreased the number of cells per focus, whereas SB203580 only caused a reduction in non-silenced cells. Moreover, the foci formed by C3G knock-down cells, particularly those expressing p38α, showed a reduction in cell-cell contacts, resulting in cell dispersion (Figure 6C), as expected from the role of C3G in adhesion and migration.

We next asked if Rap1 mediated C3G actions on foci formation. Single Rap1 knock-down led to a significant increase in the number of foci in p38α knock-down HCT116 cells, but not in cells expressing p38α (Figure 6D). Rap1 depletion also increased the number of foci in C3G knock-down cells, not only in the absence of p38α, but also in its presence. This indicates that Rap1 does not mediate C3G effects on anchorage-dependent growth.

**Figure 7:**
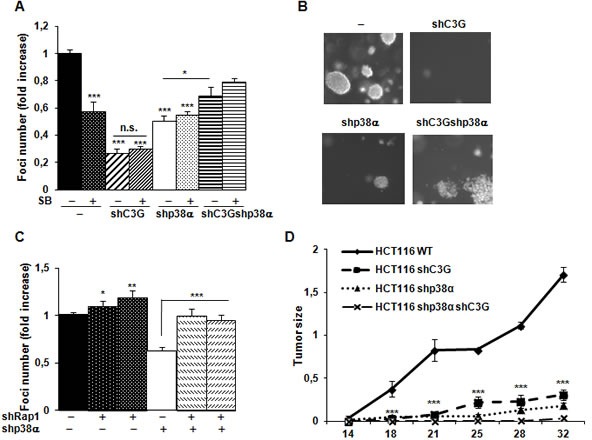
C3G and p38α MAPK promote tumor growth of HCT116 cells, while Rap1 does not HCT116 cells (non-silenced (−) and p38α knock-down (shp38α); with (shC3G) or without C3G knock-down; with (shRap1(1) and shRap1(2)) or without Rap1 knock-down) were used. **A.** and **C.** Anchorage independent growth of HCT116 cells at 14 days, in the absence or presence of SB203580 (10μM), as indicated. Histograms show the mean value ± S.E.M. of the foci number expressed as the fold increase of non-silenced cells (*n* = 4). *p < 0.05, **p < 0.01, ***p < 0.001 as compared with non-silenced cells, or as indicated. **B.** Representative images of individual foci. **D.** Xenograft assay. Immunodeficient mice were injected subcutaneously with HCT116 cells. Tumor size was calculated by the formula ((L/2)x(W/2))xπ, where L and W are the longest and the shortest diameter in centimeters, respectively. Graphs show the mean value ± S.E.M. of tumor size at the indicated time points (*n* = 6). ***p < 0.001 *versus* non-silenced cells.

Next, we analyzed the effect of C3G on anchorage-independent growth. The number of foci was highly reduced upon C3G knock-down, p38α knock-down, double C3G-p38α-knock-down or p38α inhibition with SB203580 (Figure 7A). However, the largest reduction in the number of foci was observed in C3G depleted HCT116 cells expressing p38α. Moreover, foci generated by non-silenced HCT116 cells are larger in size (Figure 7B). Curiously, C3G-p38α double knock-down cells foci seemed to have less and more dispersed cells. On the other hand, foci number was increased upon Rap1 knock-down (Figure 7C), mainly in p38α knock-down HCT116 cells, as observed in anchorage-dependent assays.

Finally, we assessed the involvement of C3G on the tumorigenic activity of HCT116 cells in nude mice. Upon subcutaneous inoculation of control HCT116 cells, tumors were readily visible at 14 days and progressively grew over time (Figure 7D). Interestingly, tumor size was significantly reduced in cells subject to either C3G or p38α knock-down. Tumor growth was almost undetectable in cells with double C3G -p38α knock-down.

**Figure 8:**
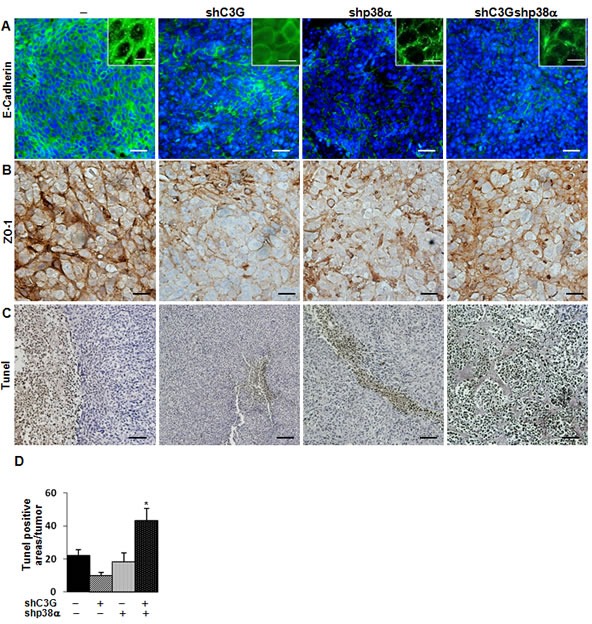
Analysis of E-cadherin, ZO-1 and cell death in tumors derived from C3G, p38α and C3G-p38α knock-down HCT116 cells End point tumors generated from HCT116 cells (non-silenced (−) and p38α knock-down (shp38α); with (shC3G) or without C3G knock-down) were analyzed. **A.** E-cadherin (green) and **B.** ZO-1 (brown) staining using specific antibodies. Inserts in **A.** show a higher amplification of images. **C.** Dead cells detected by Tunel assay (brown). **D.** Histogram showing the mean value ± S.E.M. of the percentage of Tunel positive areas per tumor. *p < 0.05 *versus* non-silenced cells. Scale bars: E-cadherin 30μm (inserts 10μm); ZO-1 20μm; Tunel assay 50 μm.

To further understand the mechanisms involved in the *in vivo* regulation of tumor growth, we analyzed the morphology of tumor cells, as well as the presence of stromal cells and dead cells within the tumors. E-cadherin (Figure 8A) and ZO-1 (Figure 8B) expression decreased in xenografts derived from C3G, p38α or C3G-p38α double silenced HCT116 cells. Additionally, E-cadherin was partially internalized in C3G knock-down cells (Figure 8A), which is consistent with the migration and invasion data. This disruption of cell-cell interactions might also contribute to the impaired tumor formation. We also found a higher number of Tunel positive cells in C3G-p38α double knock-down HCT116-derived tumors (Figure 8C and 8D), with almost 70% of apoptotic cells in the small nodules. This suggests that depletion of C3G and p38α together sensitize cells to apoptotic stimuli, ultimately resulting in the inhibition of tumor formation. In agreement with this, oxidative stress (H_2_O_2_) markedly reduced *in vitro* cell viability in these cells, with a magnitude larger than in single C3G or p38α knock-down HCT116 cells (Supplementary Figure 6). The lack of attachment also increased apoptosis in C3G-p38α double knock-down cells *in vitro*, although single C3G silencing had a greater effect (Supplementary Figure 7).

**Figure 9:**
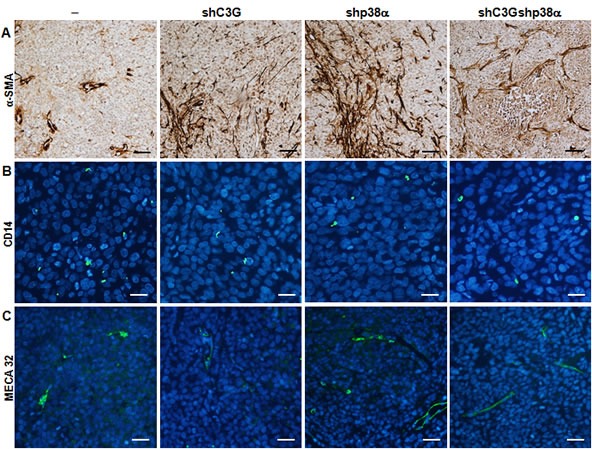
Analysis of infiltrated cells in tumors derived from C3G, p38α and C3G-p38α knock-down HCT116 cells End point tumors generated from HCT116 cells (non-silenced (−) and p38α knock-down (shp38α); with (shC3G) or without C3G knock-down) were analyzed. **A.** αSMA (brown), **B.** CD14 (green) and **C.** MECA32 (green) staining using specific antibodies. Scale bars: αSMA 50μM; CD14 20μM; MECA32 30μM.

Finally, we observed elevated fibroblast infiltration in tumors with either C3G and/or p38α silencing (Figure 9A), whereas macrophage infiltration was decreased (Figure 9B). Furthermore, angiogenesis was clearly increased upon inhibition of p38α, as revealed by the increase in vessel density using by Meca 32 staining, and this effect was independent of C3G (Figure 9C).

## DISCUSSION

The function of C3G in cell migration has not been well characterized, although a number of studies indicate that it can play distinct specific roles depending on the context [3–6, 8]. For example, C3G deficient MEFs exhibited increased migration [3–4]. Accordingly, C3G overexpression reduced cell migration of highly invasive breast carcinoma cells [11]. In contrast, C3G/Rap1 pathway mediates IGF-1-induced migration of MCF-7 breast cancer cells [43]. In this study, we demonstrate that in MEFs and HCT116 colon carcinoma cells, C3G down-regulates migration and invasion through a mechanism dependent on p38α. In fact, C3G silencing enhances migration/invasion through the up-regulation of p38α activity, which was impaired upon Rap1 silencing. Therefore, our results indicate that under physiological conditions, C3G decreases migration and invasion through a mechanism that interferes with Rap1-dependent p38α activation. This could involve specific protein-protein interactions rather than a GEF dependent mechanism [9–11, 16, 34], such as a direct C3G and p38α interaction, as observed in CML cells [34]. However, upon C3G down-regulation, other Rap1 GEFs [13, 42] would enhance Rap1 activation as a compensatory mechanism, leading to p38α hyper-activation. This is supported by the fact that Rap1-GTP levels were up-regulated in C3G-silenced MEFs under growing conditions or in response to certain stimuli such as osmotic stress (Supplementary Figure 8).

Our findings support the concept that Rap1 promotes cell migration and invasion, as previously shown in several cancer cell lines [43–49]. Accordingly, optimal cell migration was associated with cycles of Rap1 activation [49]. Additionally, our data indicate that Rap1-mediated p38α activation is required to promote migration and invasion. This agrees with the function of Rap1 as an activator of p38 in response to FGF-2 in endothelial cells [50]. However, Rap1 can also inhibit p38 activation [51] or act in a parallel pathway in other contexts [52].

Our results also support a central role for p38α in the actions of C3G on migration and invasion. We and others have previously shown that p38α plays roles in different aspects of cell migration, invasion and metastasis, favoring tumor progression [27, 37]. p38α mediates migration in HeLa cells and MEFs through the regulation of actin cytoskeleton *via* MK2 [28]. In HGF/Met-activated cortical neurons, the Rac1/p38 cascade is also crucial for migration [53]. p38α can also induce a cytoskeletal remodeling and a migratory response in tumor cells through Hsp27 phosphorylation [54]. Our results indicate that C3G, acting through p38α, regulates actin cytoskeleton organization in HCT116 cells. Thus, in C3G silenced cells, p38α hyperactivation promotes the formation of filopodia and other migratory structures. In addition, C3G depletion induces internalization of ZO-1 and partial loss of E-cadherin, which disrupts cell-cell interactions and favors cell migration. This is also supported by *in vivo* data derived from xenografts assays, which show a reduction in ZO-1 and E-cadherin expression as well as a partial internalization of E-cadherin. In MEFs, C3G down-regulation also leads to a p38α-dependent actin reorganization (data not shown) and a loss of cell-cell contacts (Suppl. Video MEFs-shC3G).

In melanoma cells [22] or ovarian cancer cells [55], p38 MAPK promotes cell migration and invasion through regulation of MMP9. p38α also induces the expression of MMP1, MMP2, MMP9 and MMP13 in other types of cancer [37]. Our results indicate that C3G silencing-mediated p38α hyperactivation increases MMP2 and MMP9 activities in MEFs and HCT116 colon carcinoma cells, which correlates with the effect on cell migration and invasion. This fits with our previous observations that p38α is a positive regulator of MMP2 and MMP9 activities [27]. Rap1 also activates MMP2 and MMP9 in cells expressing p38α, in agreement with the role of Rap1 in inducing MMP9 secretion and invasion in head and neck squamous carcinoma cells [56]. Recent published data also suggest that C3G, acting through Rap1, promotes invasion of epithelial ovarian cancer cells through induction of MMP2 and MMP9 secretion [20].

**Figure 10:**
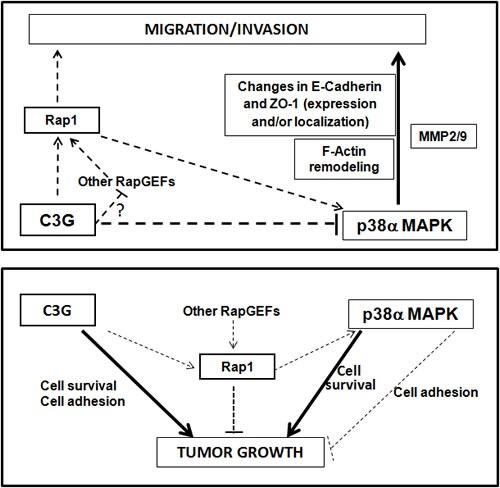
Scheme showing the interplay between C3G, p38α and Rap1 to regulate cell migration, invasion and tumor growth of HCT116 cells Upper panel, the diagram shows that C3G inhibits cell migration and invasion through down-regulation of p38α activity, either by preventing Rap1-mediated p38α activation by other Rap1GEFs or through p38α inhibition by alternative mechanisms. p38α promotes these processes. Changes in E-cadherin and ZO-1 expression and their cellular internalization, together with F-actin remodeling would mediate C3G/p38α actions on migration and invasion. MMP2 and MMP9 contribute to p38α induced invasion. Lower panel, the diagram shows that C3G and p38α promote tumor growth probably through independent pathways, inducing cell survival. Additionally, C3G through activation of cell adhesion may favor tumor growth. Rap1 inhibits tumor growth mainly through p38α independent mechanisms, although Rap1-mediated p38α activation could also prevent tumor growth by decreasing cell adhesion.

Our studies using HCT116 colon carcinoma cells also revealed a positive role for both p38α and C3G in promoting *in vitro* and *in vivo* tumor growth. Our data support the concept that C3G regulates tumor growth mainly through p38α independent mechanisms, which differs from the mechanism by which it regulates migratory and invasive responses. Additionally, Rap1 does not mediate these C3G-driven effects either, but rather counteracts them. Results from our *in vivo* studies revealed that C3G and p38α double knock-down lead to a larger reduction in tumor size relative to each individual knock-down. However, this was not observed in anchorage-dependent or -independent growth assays, where the number of foci was higher in the double knock-down than in C3G knock-down, although there were fewer cells per focus. One plausible explanation for the discrepancy between *in vitro* and *in vivo* results may be that in the *in vivo* context, other mechanisms may influence the C3G response. In fact, enhanced cell death is observed in these C3G-p38α knock-down-derived tumors, which may limit tumor development. This is also supported by the low viability detected *in vitro* in C3G-p38α silenced HCT116 cells subjected to oxidative stress (Supplementary Figure 6) or to a lack of attachment (Supplementary Figure 7). However, although the presence of dead cells in xenografts from single C3G knock-down cells at the end point was quite low, apoptosis induced *in vitro* by the loss of attachment was enhanced in these cells. This fact, together with the low adhesion of these cells, might explain the impairment in tumor cell growth *in vivo* and *in vitro*. In fact, the low adhesion observed in C3G knock-down cells correlates with a low number of foci. p38α inhibition with SB203580 or Rap1 knock-down counteracted this effect of C3G silencing on adhesion, increasing also the number of foci. Hence, Rap1-mediated p38α hyperactivation might limit cell attachment and tumor growth upon C3G depletion, although other Rap1 independent mechanisms might contribute to the reduced tumor growth (Figure 10).

Overall, our data demonstrate that C3G down-regulation promotes migration and invasion in MEFs and HCT116 colon carcinoma cells through a mechanism that requires p38α activation, and that may be mediated by Rap1 hyperactivation by other Rap1 GEFs (Figure 10). Down-regulation or inactivation of Rap1 impairs migration and invasion as a consequence of the low p38α activity. On the other hand, C3G and p38α promote growth of HCT116 cells *in vitro* and *in vivo*, most likely through different mechanisms. The effect of C3G might be dependent on its pro-adhesive and pro-survival activities (Figure 10), which might allow attachment and subsequent proliferation and/or survival of the cells. The pro-adhesive effect of C3G would be partially counteracted by Rap1-mediated p38α activation, although Rap1 would also inhibit tumor growth through other effectors. In addition, p38α might promote cell survival and/or proliferation as previously demonstrated [28, 57] through C3G-Rap1 independent mechanisms.

## MATERIALS AND METHODS

### Cell lines and cell culture

Wt and p38α−/−mouse embryonic fibroblasts (MEFs) were generated in our laboratory and immortalized by passages. C3G was permanently silenced using a C3G shRNA inserted in the pSuper.retro.puro vector [33] and cells were selected with 2μg/ml puromycin (Sigma-Aldrich P8833). To inhibit Rap1 function, MEFs (wt and p38α-deficient) expressing a Rap1 dominant-negative (with Ser17 mutated to Asn) were previously generated and selected with 2μg/ml hygromycin [33].

The human colorectal carcinoma HCT116 cell line was obtained from ATCC (CCL-247) and authenticated by microsatellite markers analysis. HCT116 cells with permanent p38α knock-down were previously generated using a p38α shRNA inserted in pSuper.retro.puro vector [58] and selected with 2μg/ml puromycin. As a control, cells transfected with the empty vector were also generated. C3G was stably knocked-down by infection with human C3G shRNAs Lentiviral Particles (75000 infectious units) containing a mixture of different shRNAs (Santa Cruz Biotechnology sc-29863-V) in the presence of 10 μg/ml Polybrene (Santa Cruz Biotechnology sc-134220) or a control shRNA for non-silenced cells. Cells were selected with puromycin (2μg/ml) and several clones were obtained, keeping those with C3G protein levels ranging between 30 and 40%. Different clones were used for the main experiments.

Rap1 was knocked-down using two different specific shRNAs (shRap1(1) and shRap1(2)) against human Rap1 (Sigma TRCN0000029784 and TRCN0000029788, respectively) by transient transfections (using Metafectene-Pro) with a plasmid carrying shRNA 1 or 2.

MEFs were grown in DMEM medium and HCT116 cells in McCoy's (Invitrogen) medium supplemented with 10% fetal bovine serum (FBS) plus antibiotics at 37°C, 5% CO_2_ in a humidified atmosphere.

p38α and/or p38β were inhibited with SB203580 (Calbiochem; 559389) at 5-10 μM.

### Cell extracts preparation and western-blot analysis

Cells were lysed in a buffer containing 50 mM Tris·HCl (pH 7.5), 150 mM NaCl, 1% NP40, 5 mM EGTA, 5 mM EDTA, 1 mM phenylmethylsulfonyl fluoride, 10 μg/ml aprotinin, 10 μg/ml leupeptin, 1 mM Na_3_VO_4_ and 20 mM NaF and centrifuged (at 13.000 rpm 10 min, 4°C). Supernatants (total cell extracts) were stored at −80°C. Protein concentration was determined by the Bradford method.

Western-blot analysis was carried out as previously described [30] using total cell extracts. Proteins were separated by electrophoresis using Anderson gels [59] (or SDS-page gels) and transferred to nitrocellulose membranes that were probed with the following antibodies against: P-p38MAPK (9211) P-ERKs (9101), P-Ser 473 Akt (9271) from Cell Signaling Technology, C3G (H-300) (sc-15359), Rap1 (sc-65), p38α MAPK (sc-535) from Santa Cruz Biotechnology, E-cadherin (BD 610182), ZO-1 (Invitrogen 617300) and β -actin (Sigma A5441).

### Wound healing assays

Confluent cells were pre-treated with mitomycin C (25 μg/ml, Sigma-Aldrich M0503) for 30 min to inhibit cell growth. Then, a straight scratch was performed and the medium replaced by a fresh one without serum (for MEFs) or with 2% FBS (for HCT116 cells). Cells were maintained for 8h-48h at 37°C and 5% CO_2_. Migration was followed by a phase-contrast microscope (Eclipse TE300 Nikon coupled to a digital camera) at different time points. Photographs were taken to quantify (using TScratch program) the percentage of wound healing closure at the different times.

### Invasion assays

Invasion was assayed using Matrigel (444 μg/cm^2^) (BD Biosciences, 356234) coated transwells (8 μm filter, BD 353097). Cells (20.000-50.000) were seeded in the upper chamber in a serum-free medium. In the lower chamber, FBS (10%) or HGF (40 ng/ml) was added to the medium to act as a chemoattractant. Then, cells were incubated for 24h at 37°C, 5% CO_2_ in a humidified atmosphere. Medium and Matrigel from the upper chamber were removed and cells present in the lower chamber were fixed with 4% paraformaldehyde and stained with crystal violet 0.2% p/v (Sigma-Aldrich C-0775). Cells were counted using a phase-contrast microscope.

### Quantification of MMP2/9 by zymography

To determine MMP2 and MMP9 activities, 80% confluent cells were serum-deprived for 24-48h and the culture medium was used for an electrophoresis in 8% SDS-polyacrylamide gels polymerized in the presence of 0.1% gelatin under non-reducing conditions. Gels were washed with 2.5%Triton X-100 (30 min) to remove SDS, rinsed with substrate buffer (0.2 M NaCl, 5 mM CaCl_2_, 1% Triton X 100, 0.02% NaN_3_, 50 mM Tris pH 7.5) and incubated in this buffer at 37°C overnight to allow protein renaturation and MMP activation. To visualize gelatin degradation, the gel was stained with Coomassie Brilliant Blue (BioRad, 161-0400).

### Adhesion assays

Trypsinized HCT116 cells were resuspended in McCoy's medium containing 10% FBS, seeded and kept in the incubator at 37°C for 1h. After washing with PBS, adhered cells were stained with Crystal violet and counted under the microscope.

### Confocal microscopy analysis

The subcellular localization of E-cadherin and ZO-1 proteins was analyzed by confocal fluorescence microscopy using the same antibodies used for western blots. Cells were seeded on 2% gelatin-coated glass coverslips and fixed with 4% paraformaldehyde at room temperature (RT) for 30 min. F-actin staining was performed using rhodamine-conjugated phalloidin as previously described [60]. To detect ZO-1, in addition to fixation, cells were permeabilized with 0.1% triton X-100 in 0.1% BSA-PBS for 20 min [60]. Fixed cells were incubated in blocking solution (2 %BSA in PBS, 1 h at RT), followed by an incubation with mouse anti-E-cadherin or rabbit anti-ZO-1 (dilution 1:50) in 0.1% BSA PBS for 1 h at RT. After washing with PBS, cells were incubated for 1 h at RT with FITC-labelled anti-mouse or anti-rabbit Alexa 594 (dilution 1:200) in 0.1% BSA PBS, respectively. After washing with PBS, cells were prepared for visualization by embedding in Vectashield mounting medium with DAPI and visualized in a Leica TCS-SL confocal microscope with a 63X objective.

### Focus formation assays

To measure anchorage dependent growth, 300 cells (HCT116 cells) were seeded in a 10 cm dish. After 8-10 days, foci were stained with a 0.2% crystal violet solution. The total number of foci was quantified using Image J program and their size using OpenCFU program. The size of colonies was measured as volume applying the equation 4/3πr3, where r is the radius of foci.

### Anchorage-independent growth in soft agar

To measure anchorage-independent growth, cells were cultured in 24-well dishes containing two agar layers. Cells (3×10^3^) were resuspended in 0.35% agar (BD, 214530) (diluted in complete medium) and poured onto a 0.5% layer of agar (diluted in medium). Fresh medium was added to the top layer every 3 days. After 2 weeks, colonies were stained with 0.005% crystal violet and counted using a dissecting microscope.

### Xenograft assays

HCT116 cells (10^6^ cells/100μl) were resuspended in McCoy's medium and injected subcutaneously (s.c.) into the flank of eight-week old male nude mice (Harlam Laboratories). Tumor growth was monitored twice a week for 6 weeks. Tumor size was calculated by the formula ((L/2)x(W/2))xπ, where L and W are the longest and the shortest diameter in centimeters, respectively. All animal experiments were carried out in compliance with the institutions guidelines.

### Immunohistochemical and immunofluorescence analysis of tumor samples

Paraffin embedded sections from HCT116 tumors were used to detect E-cadherin (cell signaling, 3195S), ZO-1 (Life technologies, 339100), MECA 32 (BD Pharmigen, 550563), CD14 (BD Pharmigen, 553739), αSMA (DAKO, M0851) and cell death by Tunel (Roche, 11093070910). For immunohistochemical analysis of ZO-1 and αSMA and detection of cell death by Tunel, endogenous peroxidase activity was first quenched. Then, binding of the primary antibody (ZO-1 and αSMA) was carried out overnight at 4°C, followed by secondary antibody incubation (1h at RT), or alternatively, for Tunel assay, incubation for 1h at 37°C with biotin 16-dUTP (Roche, 11093070910) in the reaction mixture was performed. Finally, samples were incubated with the avidin/biotin reagent (Vectastain ABC Kit) for 30 min at RT in the dark and the dAB reagent (Peroxidase Substrate Kit SK-4100). For immunofluorescence analysis of E-cadherin, MECA32 and CD14, incubation with the primary antibody (overnight at 4°C) was followed by incubation (1 h at RT) with the secondary antibodies: Alexa fluor 488 Goat anti-rabbit (Life technologies, A11034) for E-cadherin and Alexa Fluor 488 Goat anti-rat (Life technologies, A11006) for CD14 and MECA 32.

### Statistical analysis

Data are represented as the mean values ±SEM. An unpaired Student's *t*-test and Anova analysis were used.

## Supplementary Material











## ABBREVIATIONS

MAPKs: mitogen activated protein kinase
C3G: Crk SH3-domain-binding guanine nucleotide-releasing factor
MMPs: matrix metalloproteases
