# Supplementary material for: C3G knock-down enhances migration and invasion by increasing Rap1-mediated p38α activation, while it impairs tumor growth through p38α-independent mechanisms
Source: Oncotarget. 2016 Jun 7;7(29):45060–78. doi: 10.18632/oncotarget.9911 (PMC5216706; doi:10.18632/oncotarget.9911)
Supplement: Supplementary file 1 [file oncotarget-07-45060-s001.pdf]

C3G knock-down enhances migration and invasion by increasing Rap1-mediated p38α activation, while it impairs tumor growth through p38α-independent mechanisms

Supplementary Material

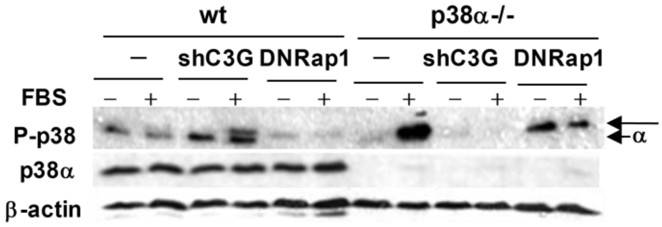

Supplemental Figure 1: Effect of C3G knock-down and DNRap1 expression on p38α MAPK activation in MEFs. Western-blot analysis of P-p38 MAPK levels normalized with β-actin. p38α was used as a control of its expression.

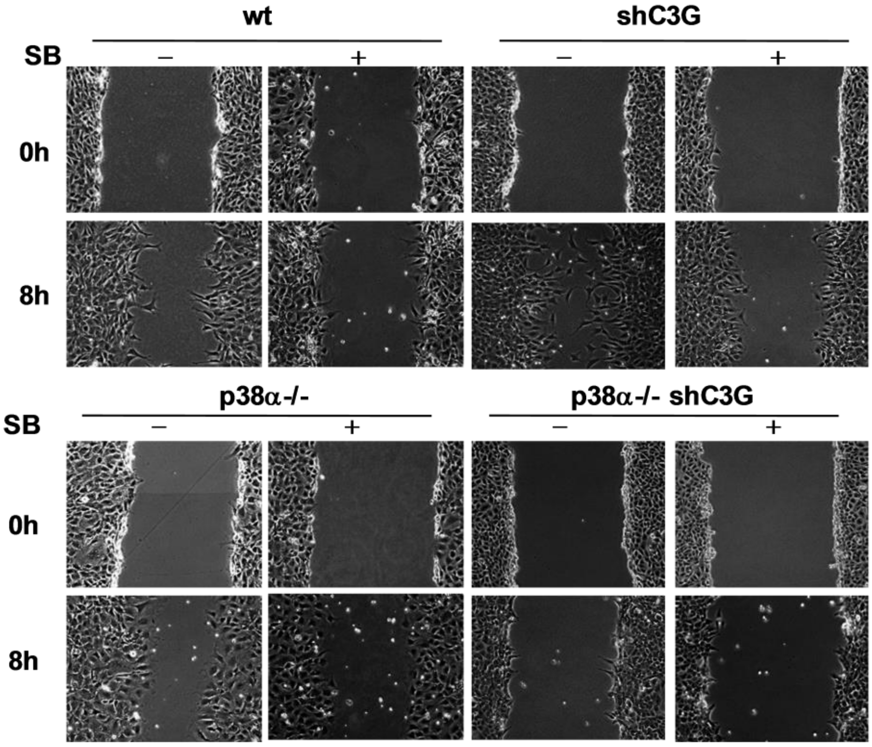

Supplemental Figure 2: p38α/β inhibition impairs the effect of C3G knock-down enhancing migration of MEFs. Wound healing assay. Effect of p38α/β inhibition with the chemical inhibitor, SB203580 (10μM).

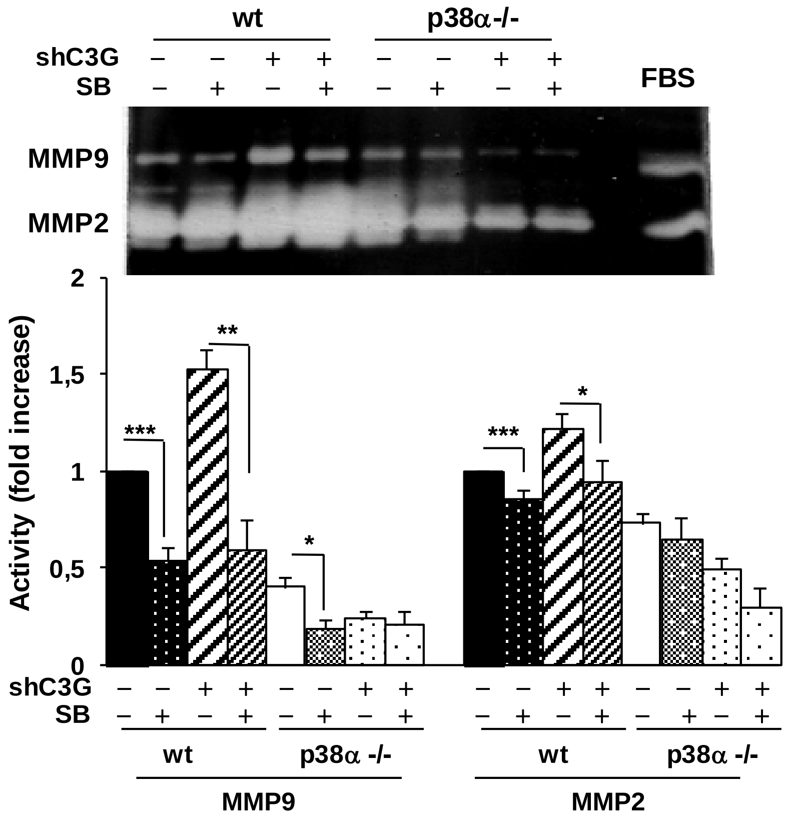

Supplemental Figure 3: C3G knock-down increases MMP2 and MMP9 activities in MEFs. Effect of p38α/β inhibition. Zymographic analysis of MMP2 and MMP9 activities using gelatin as the substrate and FBS as a control. Representative zymogram (upper panel). Histogram (lower panel) shows the mean ± S.E.M. of the densitometric analysis of gelatinase areas expressed as fold increase of the control value (n=6). *p<0.05, **p<0.01,***p<0.001 SB treated versus untreated cells. (E) Effect of p38α/β inhibition with SB203580 (10μM).

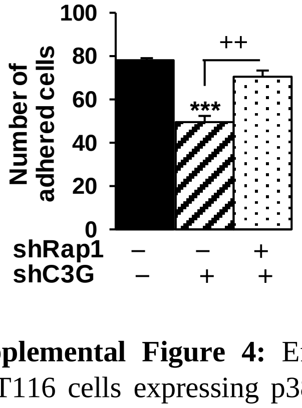

Supplemental Figure 4: Effect of C3G and C3G-Rap1 double knock-down on adhesion of HCT116 cells expressing p38α MAPK. Histogram shows the mean ± S.E.M. of the number of adhered cells.

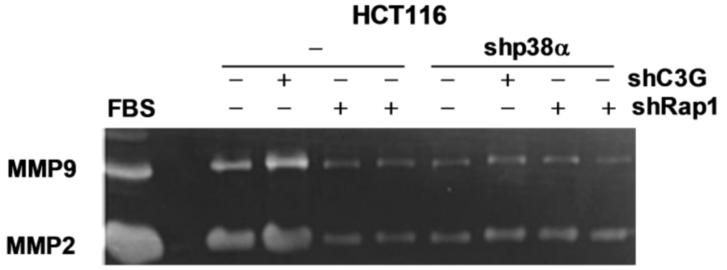

Supplemental Figure 5: Differential effect of C3G and Rap1 knock-down on MMP2 and MMP9 activities in HCT116 cells. Zymographic analysis of MMP2 and MMP9 activities using gelatin as the substrate and FBS as a control. Representative zymogram.

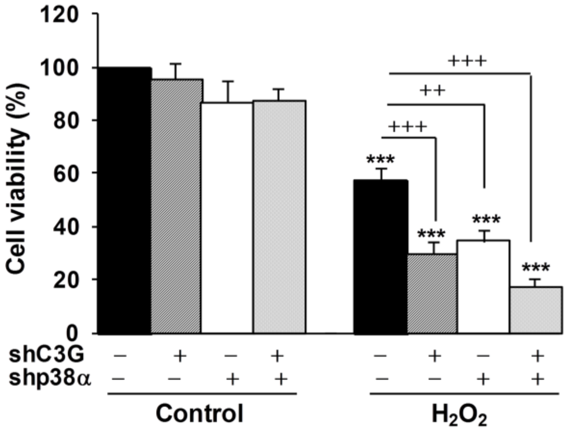

Supplemental Figure 6: Effect of C3G and/or p38α knock-down on H_2O_2-induced cell death in HCT116 cells. Cells were maintained in the absence of serum untreated or treated with 2mM H_2O_2 for 4h. Then, cell viability was determined. Histogram showing the mean value ± S.E.M. of the percentage of viable cells referred to untreated parental cells (100%) (n=3). ***p<0.001, versus non-silenced cells; $^{++}$p<0.01,$^{+++}$p<0.001, compared as indicated.

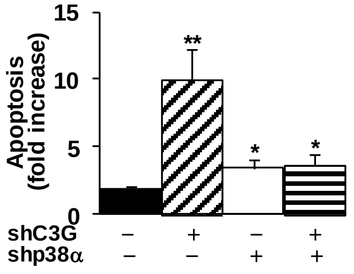

Supplemental Figure 7: Effect of the lack of attachment on apoptosis in HCT116 cells depleted of C3G and/or p38α. Cells were maintained in suspension for 6h and then, the percentage of apoptotic cells was determined by flow cytometry. Results are expressed as the fold increase of apoptosis in cells maintained in suspension as compared with those maintained attached from the same genotype. *p<0.05, **p<0.01 versus non-silenced cells.

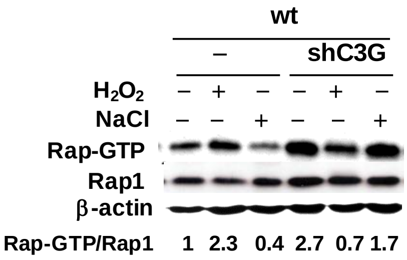

Supplemental Figure 8: Effect of C3G knock-down on Rap-GTP levels in MEFs. Cells were maintained in a medium supplemented with 10% fetal bovine serum, either untreated or treated with 0.5 mM H_2O_2 or 0.5M NaCl for 20 min. Rap-GTP levels were normalized by determining the ratio between Rap-GTP and total Rap protein levels normalized with β-actin. Rap-GTP/Rap ratio data were expressed as the fold increase of non-silenced C3G untreated cells.

Supplemental Methods

Analysis of cell viability and apoptosis

Cell viability was assayed through staining of adhered (viable) cells with crystal violet. Cells were washed with PBS, incubated with a crystal violet solution (0.2%, w/v) for 20 min, washed and dried. Stained cells were lysed in 1% SDS and absorbance at 560nm was measured.

Apoptotic cells were quantified by flow cytometric analysis of the cell cycle. Cells were trypsinized, washed with PBS and fixed with cold ethanol (70% v/v). Then, cells were washed, resuspended in PBS and incubated with RNAase (25 $\mu g/10^6$ cells) for 30 min at 37ºC. After addition of 0.05% propidium iodide (PI), cells were analyzed in the cytometer. The percentage of cells in the different phases of cell cycle was determined. Cells in subG1 were considered as apoptotic.

Analysis of Rap1 GTP levels

Rap-1 activity assay was performed with total cell lysates from MEFs using pGST-RalGDS-RBD construct to pull down Rap-1-GTP, which was analyzed by Western-blot as described previously (Gutiérrez-Uzquiza et al., Cell Signal 2010; 22: 533-542).

Supplemental Videos: Time lapse microscopy videos of MEFs during would healing closure.

-Video 1-wt MEFs
-Video 2-shC3Gwt MEFs (C3G knock-down wt MEFs)
-Video 3-p38α-/- MEFs
-Video 4-shC3Gp38α-/- MEFs (C3G knock-down p38α knock-out MEFs)
